# Proteomic Evaluation of Acquired Enamel Pellicle during *In Vivo* Formation

**DOI:** 10.1371/journal.pone.0067919

**Published:** 2013-07-03

**Authors:** Young Ho Lee, Jason N. Zimmerman, William Custodio, Yizhi Xiao, Tayebeh Basiri, Sahza Hatibovic-Kofman, Walter L. Siqueira

**Affiliations:** Departments of Biochemistry and School of Dentistry, Schulich School of Medicine and Dentistry, The University of Western Ontario, London, Ontario, Canada; University of Technology Sydney, Australia

## Abstract

Acquired enamel pellicle (AEP) is a protein film that forms on the enamel surface of teeth by selective adsorption of proteins and peptides present in the mouth. This protein film forms the interface between enamel and the damage oral biofilm, which modulates the attachment of bacteria found in oral biofilm. The overall goal of this study was to gain insight into the biological formation of the human *in vivo* AEP. This study hypothesized that AEP is created by the formation of successive protein layers, which consist of initial binding to enamel and subsequent protein-protein interactions. This hypothesis was examined by observing quantitative and qualitative changes in pellicle composition during the first two hours of AEP formation in the oral cavity. Quantitative mass spectrometry approaches were used to generate an AEP protein profile for each time-point studied. Relative proteomic quantification was carried out for the 50 proteins observed in all four time-points. Notably, the abundance of important salivary proteins, such as histatin 1, decrease with increasing of the AEP formation, while other essential proteins such as statherin showed constant relative abundance in all time-points. In summary, this is the first study that investigates the dynamic process to the AEP formation by using proteomic approaches. Our data demonstrated that there are significant qualitative and quantitative proteome changes during the AEP formation, which in turn will likely impact the development of oral biofilms.

## Introduction

The composition of the acquired enamel pellicle (AEP) formed *in vivo* has been studied by many techniques, including microscopy [1], amino acid analysis [2], [3], gel-filtration and ion-exchange chromatography [4], [5], and electrophoresis and immunoblotting [6], [7], [8], [9], [10], [11], [12]. All of these studies have been limited by the difficulties encountered in obtaining adequate amounts of AEP material for classical biochemical characterization [13]. However, a consistent finding has been that the amino acid compositions of pellicles from different subjects are remarkably similar [3]. Recent developments of sensitive proteomic methodologies have opened new avenues for the characterization of very-low-abundance biological samples such as AEP. Using this proteomic technology, studies have been carried out to explore the composition of *in vitro* [14], [15], *in situ* [16], [17] and *in vivo* AEP [15], [18]. As a results of this novel technology, our group have used mass spectrometry to perform the first global proteome of human pellicle [19]. We have successfully identified 130 AEP proteins, which have been characterized according to origin, putative biological function and possible role in AEP structure. A surprising finding was that only 14% of the identified proteins were derived from exocrine salivary secretions. Most of the identified pellicle proteins originated from the non-exocrine contributors to whole saliva, comprising epithelial cells (68%) and serum (18%). The latter oral fluid contributor enters the oral cavity through the gingival crevice.

When the 130 proteins were categorized based on their possible role in AEP formation, three main groups, together adding up to 61% of all proteins were identified. The first group consist of proteins that have the ability to bind calcium ions, comprising 18% of the identified AEP proteins. Among these are the acidic PRPs and histatins, both proteins originating from exocrine salivary secretions. The second group (15%) consists of proteins that show a high tendency to bind phosphate ions, such as elongation factor 2 and myosin-9, both proteins are derived from epithelial cells. The third group (28%) consists of proteins that have been described to have interactions with other proteins. An example is MUC5B, which has been described to form complexes with several other salivary proteins, including salivary α-amylase, histatin and statherin [20], [21].

We hypothesize that *in vivo* AEP pellicle is created by the formation of successive protein layers, based initially on binding to tooth mineral (calcium and phosphate) and subsequently on protein-protein interaction. This working hypothesis will be examined by assessing quantitative and qualitative changes in AEP composition during the first two hours of formation in the oral cavity. Quantitative mass spectrometry approach will be performed to generate an AEP protein profile for each time-point studied. We expect to identify and characterize the particular proteome profiles for the initial and final stages of AEP formation, where proteins or peptides with affinity to hydroxyapatite will be more abundant in the first stages of AEP formation, and the remaining AEP components will be subsequently incorporated into the pellicle film.

## Methods

### Acquired Enamel Pellicle Collection

This study was approved by the Research Human Ethics Board of the University of Western Ontario (review number 16181E). Written informed consent was acquired from all subjects in this study. AEP were collected from 7 patients including 4 male and 3 female (aged 20 to 30 years old). The participants were healthy individuals who did not have systemic and oral diseases. The samples were collected between 9 am and 11 am. Subjects were not allowed to eat or drink 2 hours before the sample collection. Each participant was subjected to dental prophylaxis in order to remove the previous existing AEP. Subsequently, they were asked to wait for each time-points in order for AEP to form on the enamel surface. Four different time-points were used in the current study; including 5, 10, 60, and 120 min. The collections were carried out as described before [22] on different days for each time-point using the same volunteers. After collecting, the samples were kept at –80°C.

### AEP Elution from Collection Strips via Sonication

All collection strips were pooled into a 15 mL Falcon tube. Pool samples from each time-point were kept separately. Three mL of 50 mM NH_4_HCO_3_, pH 7.8 were added into the tubes until all the strips were submerged by the solution. Subsequently, the samples were sonicated at room temperature for 1 min. The supernatants were collected and dried in a rotary evaporator. Micro-BCA was performed to measure the total protein concentration from each AEP time-point.

### In–Solution Digestion

Equal protein amount (20 µg) from each time-point group was dried by a rotary evaporator, denatured and reduced for 2 h by the addition of 200 µl of 4 M urea, 10 mM dithiothreitol (DTT), and 50 mM NH_4_HCO_3_, pH 7.8. After four-fold dilution with 50 mM NH_4_HCO_3_, pH 7.8, tryptic digestion was carried overnight at 37°C, after the addition of 2% (w/w) sequencing-grade trypsin (Promega, Madison, WI, USA). After protein digestion period the samples were completely dried to stop the enzymatic reaction.

### Liquid Chromatography Electrospray Ionization Tandem Mass Spectrometry (LC-ESI-MS/MS)

Peptide separation and mass spectrometric analyses were carried out with a nano-HPLC Proxeon (Thermo Scientific, San Jose, CA, USA) which allows in-line liquid chromatography with the capillary column, 75 µm X 10 cm (Pico Tip™ EMITTER, New Objective, Woburn, MA) packed in-house using Magic C18 resin of 3 µm diameter and 200 Å pores size (Michrom BioResources, Auburn, CA) linked to mass spectrometer (LTQ-Velos, Thermo Scientific, San Jose, CA, USA) using an electrospray ionization in a survey scan in the range of m/z values 390–2000 tandem MS/MS. Equal amount of all samples (20 µg/each group) were re-suspended in 20 µl of 97.5% H_2_O/2.4% acetonitrile/0.1% formic acid and then subjected to reversed-phase LC-ESI-MS/MS. The nano-flow reversed-phase HPLC was developed with linear 80 minutes gradient ranging from 5% to 55% of solvent B in 65 minutes (97.5% acetonitrile, 0.1% formic acid) at a flow rate of 300 nl/min with a maximum pressure of 280 bar. Electrospray voltage and the temperature of the ion transfer capillary were 1.8 kV and 250°C respectively. Each survey scan (MS) was followed by automated sequential selection of seven peptides for CID, with dynamic exclusion of the previously selected ions.

The obtained MS/MS spectra were searched against human protein databases (Swiss Prot and TrEMBL, Swiss Institute of Bioinformatics, Geneva, Switzerland, http://ca.expasy.org/sprot/) using SEQUEST algorithm in Proteome Discoverer 1.3 software (Thermo Scientific, San Jose, CA, USA). Search results were filtered for a False Discovery rate of 1% employing a decoy search strategy utilizing a reverse database. An additional inclusion criterion for positive identification of proteins was the same protein passing the filter score from at least in three different MS analyses from the same time-point group in a total of four MS analyses per group.

### Integration and Relative Proteome Quantitation

For quantitative proteome analysis, three MS raw files from each pooled group were analyzed using SIEVE technology (Version 2.0 Thermo Scientific, San Jose, CA, USA). Signal processing was performed in a total of 12 MS raw files. The SIEVE experimental workflow was defined as “Control Compare Trend Analysis” where one class of samples was compared to one or more other class of samples. Here the control samples (5-min AEP period) were compared to each of the samples that were harvested in different time-point (10, 60 and 120 min). For the alignment step, a single MS raw file belonging to the 5-min AEP group was selected as the reference file and all of the other files were adjusted to generate the best correlation to this reference file. After alignment, the feature detection and integration (or framing) process was performed using the MS level data with a feature called “Frames From MS2 Scans” only. When using this type of framing only MS mass-to-charge ratio (m/z) values that were associated with MS2 scan were used. Any m/z measurements that did not have MS2 were ignored. The parameters used consisted of a frame m/z width of 1500 ppm and a retention time width of 1.75 min. A total of 73456 MS2 scans were present in all of the 12 RAW files that resulted in a total of 11151 frames. Then peak integration was performed for each frame and these values were used for statistic analysis. Next, peptide sequences obtained from the database search using SEQUEST algorithm in Proteome Discoverer 1.3 were imported into SIEVE. A filter was applied to the peptide sequences during the import that eliminated all sequences with a Percolator q-value greater than 1% (false discovery rate). Peptides were grouped into proteins and a protein ratio and p-value were calculated. SIEVE uses a weighted average of the peptide intensities for the protein calculation. By using the weighted average, peptides with lower variance in their intensity measurements have a higher weight on the overall protein ratio. This was done to decrease variance in protein level quantities based on variance of the peptides that compose proteins. Only proteins observed in all four time-point groups were quantified. 5-min AEP group was used as our default group and all other three groups were compared with 5-min AEP group.

Relative abundance of an individual protein from 5-min AEP group was considered significantly different protein level when the values observed were <0.75 for decrease abundance or >1.25 for increase abundance, and a *p*-value <0.05 as described [23].

## Results

**Figure 1:**
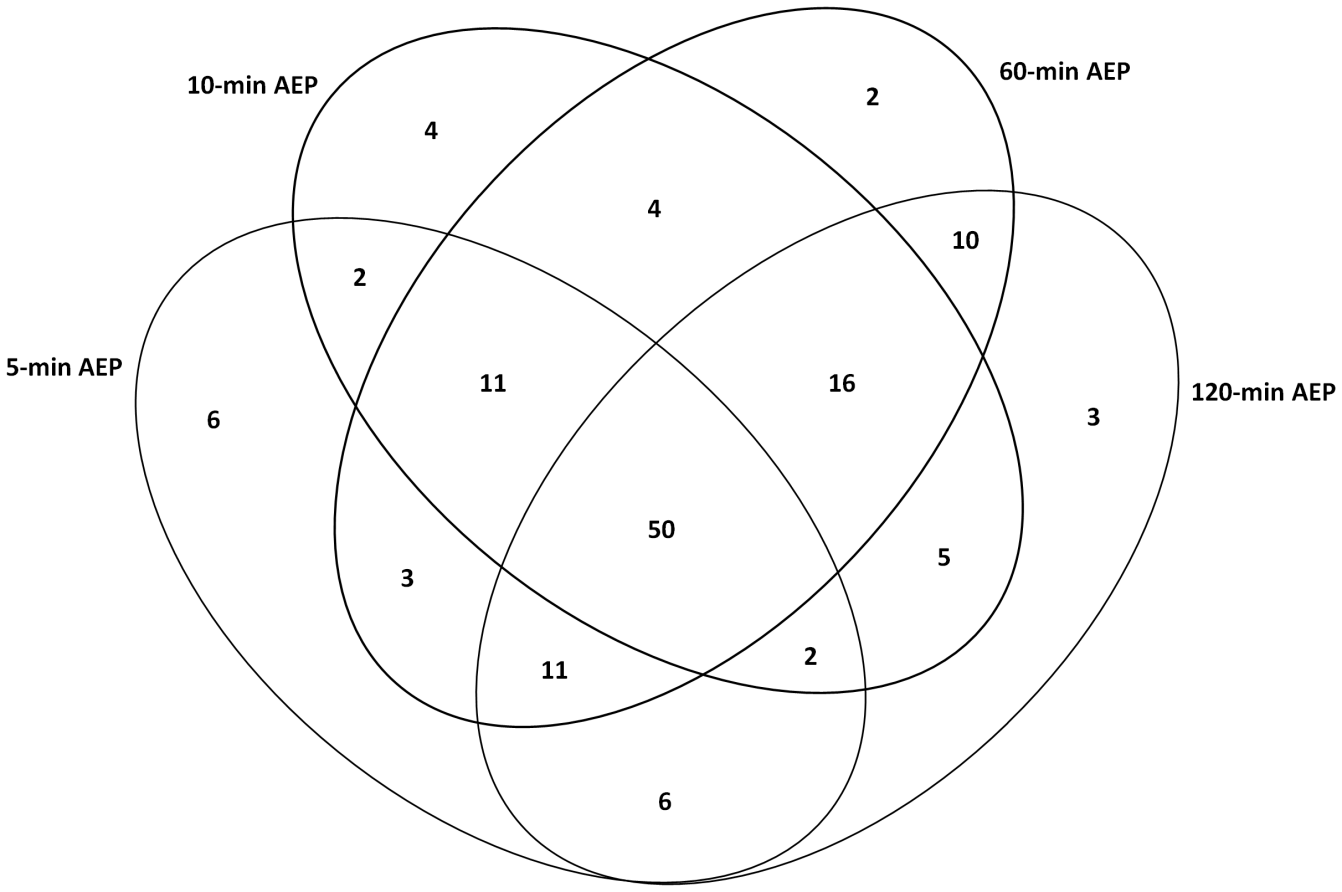
Venn diagram of AEP proteins identified in each time-point and across time-point.

The peptide ions were identified by the SEQUEST search following the criteria as described in Methods. For the proteome identification of the AEP, formed in all four different time-points carried out in this study, a total of 89 different proteins were identified in 5-min AEP formation, 92 different proteins were identified in 10-min AEP formation, 107 different proteins were identified in 60-min AEP formation and 101 different proteins were identified in 120-min AEP formation (Table 1). The majority of the proteins were identified in all four groups indicating a high overlap in AEP proteins. Figure 1 shows a Venn diagram with the number of proteins from each group and their overlaps among the four groups. A total of 50 proteins were present in all four groups. Six proteins were exclusively present in 5-min AEP group. Four proteins were exclusively present in 10-min AEP group. Two proteins were exclusively present in 60-min AEP group and other 3 proteins were present only in 120-min AEP (Table 1; Figure 1).

**Table 1:** *In vivo* AEP proteins identified after 5, 10, 60 and 120-min pellicle formation.

	Accession Number	Protein Name	Chemical properties*	
Proteins present at all AEP time-points	P23280	Carbonic anhydrase 6	Ca^+2^ and PO_4_ binding	
	Q9HCY8	Protein S100-A14	Ca^+2^ binding	
	P35527	Keratin type I cytoskeletal 9	protein-protein interaction	
	B4DRY0	cDNA FLJ54379 highly similar to Keratin type II cytoskeletal 6E	protein-protein interaction	
	A8K2I0	cDNA FLJ78504 highly similar to Homo sapiens keratin 6A (KRT6A) mRNA		
	P13645	Keratin type I cytoskeletal 10	protein-protein interaction, Ca+2 and PO4 binding	
	C3PTT6	Pancreatic adenocarcinoma upregulated factor	protein-protein interaction	
	B4DKJ0	cDNA FLJ58539 highly similar to Keratin type II cytoskeletal 4	protein-protein interaction	
	P06733	Alpha- Enolase	Ca^+2^ and PO_4_ binding	
	B7WNW7	HEAT repeat containing protein 3	protein-protein interaction, Ca+2 and PO4 binding	
	P98088	Mucin 5A	protein-protein interaction	
	P13646	Keratin, type I cytoskeletal 13		
	F8VV32	Lysozyme C	protein-protein interaction, Ca+2 binding	
	B4DWU0	cDNA FLJ56791 highly similar to Keratin type I cytoskeletal 16	protein-protein interaction	
	P02812	Basic salivary proline rich protein 2	protein-protein interaction	
	P01034	Cystatin C	protein-protein interaction, Ca+2 binding	
	P23284	Peptidyl prolyl cis trans isomerase B	protein-protein interaction	
	Q01546	Keratin type II cytoskeletal 2 oral		
	Q4VAY2	PRB3 protein	protein-protein interaction	
	Q6GMW3	IGL protein	protein-protein interaction, Ca+2 binding	
	P27482	Calmodulin-like protein 3	Ca^+2^ binding	
	P02787	Serotransferin	Ca^+2^ and PO_4_ binding	
	B4DDZ2	cDNA FLJ60419 highly similar to R3H domain containing protein 2		
	P28325	Cystatin D	protein-protein interaction, Ca+2 binding	
	P15516	Histatin 3	Ca+2 binding	
	P06702	Calgranulin B	Ca^+2^ binding	
	P02808	Statherin	Ca^+2^ binding	
	Q5BQ95	Kallikrein 13 splice variant 7		
	Q9UGM3	Deleted in malignant brain tumors 1 protein(glycoprotein 340)	protein-protein interaction	
	B4DYU5	cDNA FLJ50396 highly similar to Zinc finger and BTB domain containing protein 17		
	P04745	Alpha-amylase 1	protein-protein interaction, Ca+2 binding	
	P02647	Apolipoprotein A I	Ca+2 binding	
	Q8IWA8	PCSK2 protein		
	Q9C0E4	Glutamate receptor interacting protein 2	protein-protein interaction, Ca+2 binding	
	Q9P273	Teneurin 3		
	P35908	Keratin type II cytoskeletal 2 epidermal	protein-protein interaction, Ca+2 binding	
	P20061	Transcobalamin 1		
	F8W6D7	Protein CBFA2T2		
	P15515	Histatin 1	Ca+2 binding	
	P0C091	FRAS1 related extracellular matrix protein 3		
	F8W1S1	Keratin type II cytoskeletal 74		
	P09228	Cystatin SA	Ca+2 binding	
	B4DF17	cDNA FLJ59187 highly similar to Multiple coagulation factor deficiency protein 2 homolog		
	P02768	Serum albumin	protein-protein interaction	
	P12273	Prolactin inducible protein	protein-protein interaction	
	P01036	Cystatin S	Ca+2 binding	
	H0Y3I2	Lactoperoxidase	protein-protein interaction	
	Q9UP60	SNC73 protein		
	P01833	Polymeric immunoglobulin receptor	protein-protein interaction	
	P02810	Salivary acidic proline rich phosphoprotein 1/2	Ca^+2^ binding	
Proteins present at 5-min, 10-min and 60-min AEP time-points	P78333	Glypican-5		
	O75376	Nuclear receptor corepressor 1		
	Q9BZZ2	Sialoadhesin	protein-protein interaction	
	A7MCY6	TANK-binding kinase 1-binding protein 1	protein-protein interaction	
	Q9Y3R5	Protein dopey-2		
	B3V0L1	ADP-ribosylation factor-like protein 6-interacting protein 4		
	P20061	Transcobalamin-1		
	B4DH81	cDNA FLJ61250, highly similar to Homo sapiens GTPase activating Rap/RanGAP domain-like 3		
	Q9BTC0	Death-inducer obliterator 1		
	E7EX20	Tubulin polyglutamylase TTLL4		
	O00444	Serine/threonine-protein kinase	Ca+2 binding	
Proteins present at 5-min, 10-min and 120-min AEP time-points	P30622	CAP-Gly domain-containing linker protein 1		
	Q75T53	Putative uncharacterized protein		
Proteins exclusively present at 5-min AEP time-point	P01037	Cystatin-SN	Ca+2 binding	
	Q5T3N0	Annexin (Fragment)	Ca+2 and PO4 binding	
	P07339	Cathepsin D	protein-protein interaction, Ca+2 binding	
	Q9UBC9	Small proline-rich protein 3	Ca+2 binding	
	P04080	Cystatin-B	Ca+2 binding	
	P31949	calgizzarin(s-100calcium binding protein a11)	Ca+2 binding	
Proteins present at 5-min and 10-min AEP time-points	Q9NZU7	Calcium-binding protein 1	Ca+2 binding	
	P02679	Fibrinogen gamma chain	protein-protein interaction	
Proteins present at 5-min and 60-min AEP time-points	P62158	Calmodulin	Ca+2 binding	
	Q06830	Peroxiredoxin-1	protein-protein interaction, Ca+2 binding	
	O75828	carbonyl reductase		
Proteins present at 5-min and 120-min AEP time-points	Q8TAX7	Mucin-7	protein-protein interaction	
	P02766	Transthyretin		
	P18135	Ig kappa chain V-III region HAH	protein-protein interaction	
	Q9Y6R7	IgGFc-binding protein	protein-protein interaction	
	A1L4B8	Myeloperoxidase	Ca+2 and PO4 binding	
	P34931	Heat shock 70 kDa protein 1L		
Proteins present at 5-min, 60-min and 120-min AEP time-points	Q96S07	Proline-rich protein 25	Ca+2 binding	
	P05109	Protein S100-A8	Ca+2 binding	
	Q7Z2U7	Putative uncharacterized protein		
	Q6GMV8	Putative uncharacterized protein		
	P02765	Alpha-2-HS-glycoprotein	protein-protein interaction	
	P17213	bacterial permeability-increasing protein	protein-protein interaction	
	P11021	endoplasmic reticulum lumenal Ca binding protein	Ca+2 binding	
	P01023	Alpha-2-macroglobulin	protein-protein interaction	
	P08311	Cathepsin G	protein-protein interaction, Ca+2 binding	
	P54108	Cysteine-rich secretory protein 3		
	Q01469	Fatty acid-binding protein	protein-protein interaction	
Proteins exclusively present in 10-min AEP time-point	Q9Y6R7	IgGFc-binding protein	protein-protein interaction	
	P20930	Filaggrin	Ca+2 binding	
	Q96DR5	parotid secretory protein	Ca+2 binding	
	O43240	Kallikrein-10	Ca+2 binding	
Proteins present at 10-min and 60-min AEP time-points	P10599	Thioredoxin		
	P09211	Glutathione S-transferase P		
	P80511	calgranulin C	Ca+2 binding	
	P06703	S100 calcium-binding protein A6	Ca+2 binding	
Proteins present at 10-min and 120-min AEP time-points	B5ME49	Mucin-16	protein-protein interaction	
	P01009	Alpha-1-antitrypsin		
	P06733	Alpha-enolase	protein-protein interaction, Ca+2 binding	
	P00738	Haptoglobin		
	Q96FQ6	Protein S100-A16	Ca+2 binding	
Proteins present at 10-min, 60-min and 120-min AEP time-points	F6KPG5	Albumin (Fragment)	protein-protein interaction	
	H7C4X9	Protein kinase C-binding protein 1 (Fragment)	Ca+2 binding	
	P10161	Basic salivary proline-rich protein 4 allele M (Fragment)	protein-protein interaction	
	P04406	Glyceraldehyde-3-phosphate dehydrogenase	protein-protein interaction	
	Q6GMX1	Immunoglobulin heavy variable 4–31	protein-protein interaction	
	P68871	Hemoglobin subunit beta	protein-protein interaction	
	Q04118	Basic salivary proline-rich protein 3	protein-protein interaction	
	P25311	zinc-alpha-2-glycoprotein precursor	protein-protein interaction, Ca+2 binding	
	P04280	Basic salivary proline-rich protein 1	protein-protein interaction	
	P02812	Basic salivary proline-rich protein 2 (Fragment)	protein-protein interaction	
	P49913	Cathelicidin antimicrobial peptide	protein-protein interaction, Ca+2 binding	
	P28676	Grancalcin	Ca+2 binding	
	P11021	endoplasmatic reticulum lumenal Ca binding protein	Ca+2 binding	
	P63104	protein kinase C inhibitor protein 1	Ca+2 binding	
	P02675	Fibrinogen beta chain precursor	protein-protein interaction	
	P01876	Ig alpha-1 chain c region	protein-protein interaction	
Proteins exclusively present at 60-min AEP time-point	D3DP16	Fibrinogen gamma chain, isoform CRA_a		
	B3W5Y6	Squamous cell carcinoma antigen-1 isoform SCCA-PD		
Proteins present at 60-min and 120-min AEP time-points	Q6WRI0	Immunoglobulin superfamily member 10	protein-protein interaction	
	P04083	Annexin A1	protein-protein interaction	
	P62736	Actin, aortic smooth muscle		
	P01024	Complement C3	protein-protein interaction	
	A5D903	PRB1 protein	protein-protein interaction	
	Q96DR5	Short palate, lung and nasal epithelium carcinoma-associated protein 2	protein-protein interaction, Ca+2 binding	
	Q14568	Putative heat shock protein HSP 90-alpha A2	PO_4_ binding	
	E7EUT5	Glyceraldehyde-3-phosphate dehydrogenase	protein-protein interaction	
	F5H308	L-lactate dehydrogenase		
	P01834	Ig kappa chain c	protein-protein interaction	
Proteins exclusively present at 120-min AEP time-point	P02788	Lactotransferrin	protein-protein interaction, Ca+2 and PO4 binding	
	P32926	Desmoglein-3	Ca+2 and PO4 binding	
	P12035	Keratin, type II cytoskeletal 3	protein-protein interaction	

* Sorting of the chemical properties of these proteins was based on their annotations in the UniProt protein database (www.uniprot.org) and EMBL-EBI database (www.ebi.ac.uk).

Relative proteomic quantification was carried out in the 50 proteins observed in all AEP time-point groups. Differential display of MS/MS spectra was carried out using SIEVE software. A first step in the quantitative proteomic analysis by SIEVE was to promote an alignment of all mass spectrometry chromatograms. One of the mass spectrometry chromatogram was noted as default chromatogram (5-min AEP). All other chromatograms were compared with the default one. Coefficient correlation score values were acquired for each mass spectrometric chromatogram and mean score values were calculated for each group. The values were 0.831 to 10-min AEP group, 0.851 to 60-min AEP group and, 0.813 to 120-min AEP.

A threshold for significant differential level was set up at variation higher or lower than the 25% protein level observed in the control group. A total of 40 proteins showed a differential level between 5-min AEP group and 10-min AEP group where 22 showed a decrease level and 18 proteins showed an increase level. In addition, 19 proteins showed a decrease level between 5-min AEP group and 60-min AEP group and 17 proteins showed an increase level. Comparison of the 5-min AEP group with 120-min AEP group, demonstrated 24 proteins with a reduced protein level while 14 protein showed an increase (Table 2).

**Figure 2:**
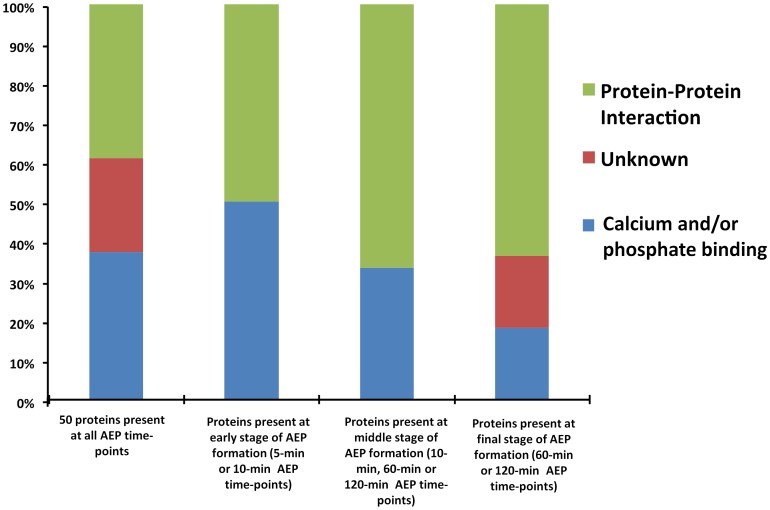
Distribution of identified proteins according to their role in AEP structure formation or molecular interaction.

Pellicle proteins for each time-point were analyzed according to their role in AEP structure formation or molecular interaction; and the proteins were segregated into three main groups (Table 1; Figure 2). Overall, AEP proteins containing calcium and phosphate binding property were more predominant when the time-points 5-min and 10-min AEP formation was counted, between 50% and 85%, respectively (Table 1). While AEP proteins with protein-protein interaction property demonstrated a gradual increment according to the pellicle development (Figure 2).

## Discussion

One of the major achievements of this study was to obtain a broader insight into the protein pattern profile of the AEP during its first two hours formation. In this study, a state-of-the-art approach, label-free quantitative proteomics based on mass spectrometry, was used to investigate the precursor protein members present in the AEP and its behavior during the AEP formation. Interestingly, according to the pellicle development was happening, the measured alignment value was become more distant than the default chromatogram (5-min AEP, established score 1). This observation suggests a change in quantity and quality of protein/peptides according to the pellicle formation.

**Figure 3:**
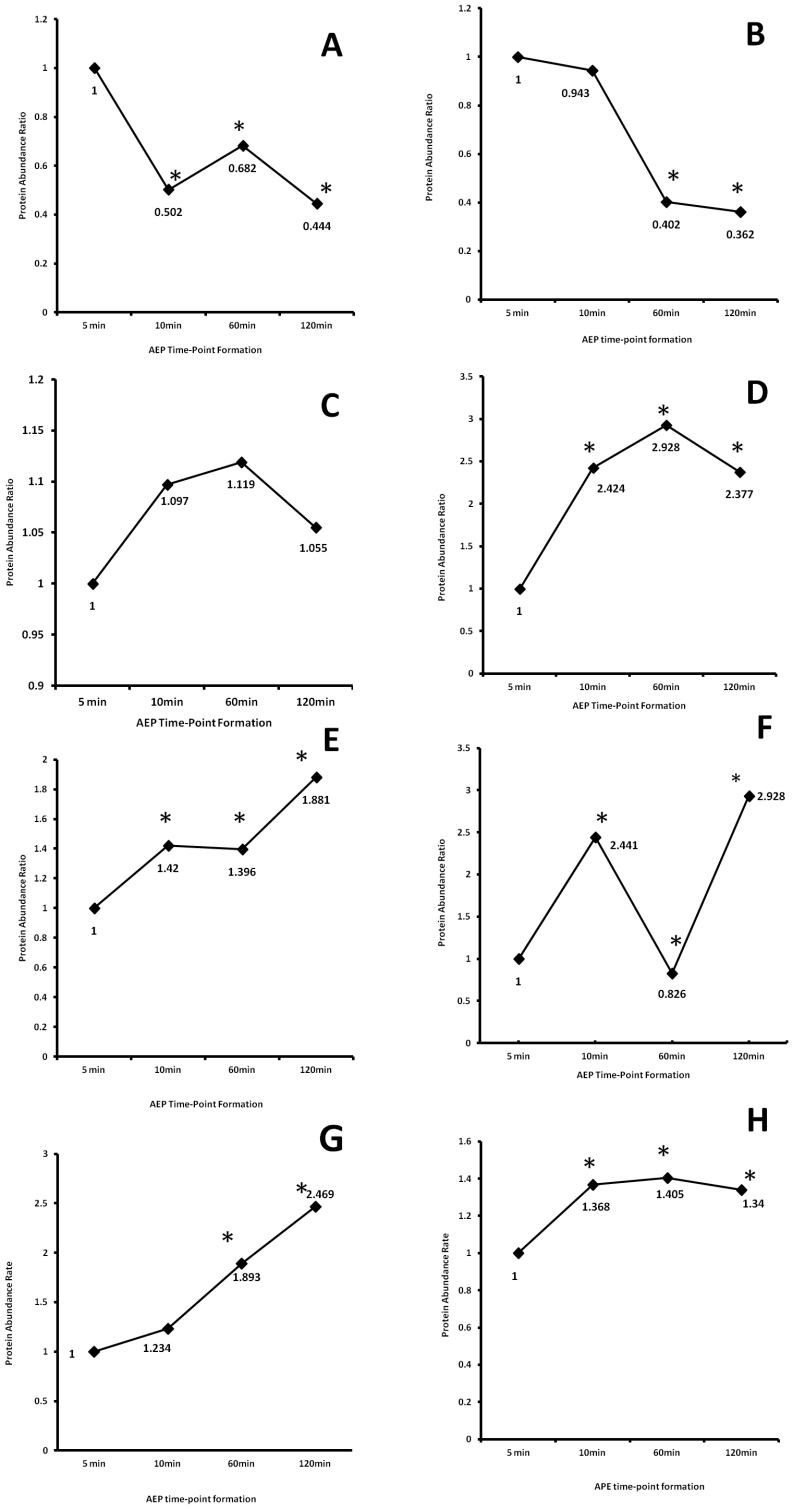
Time course of changes in the abundance of specific AEP proteins. (A) histatin 1, (B) histatin 3, (C) statherin, (D) acidic PRP1, (E) amylase, (F) MUC5B, (G) lysozyme, (H) lactoperoxidase. Note: * denotes statistical difference when compared with 5-min AEP time-point. p>0.05.

Both, histatin 1 and histatin 3 proteins demonstrated drastic reduction abundance after 60 and 120 minutes pellicle formation when compared with the first 5 minutes pellicle development (Figure 3A, B). Despite the high affinity of those proteins to the enamel surface, histatins are highly susceptible to proteolytic degradation [24], [25]. On the other hand, recent study has showed that histatin 1 when attached to the enamel surface; this protein is less susceptible to proteolytic degradation [26]. In our study, we observed a high abundance of these proteins in the initial stage of the pellicle formation (5-min AEP group), which can be correlated to the well-characterized features of these proteins to be the precursor protein in the formation of the AEP, but a significant reduction according to the time development of AEP. This observation can be related to the protein degradation susceptibility common observed in this protein family when in saliva or attached to the enamel surface.

Other important salivary proteins such as statherin, that presents similar characteristics as histatins, has high affinity to hydroxyapatite and susceptible to proteolytic degradation when in saliva [27], has demonstrated a complete different pattern. Statherin relative protein abundance did not change during the time period for *in vivo* AEP development (Figure 3C). This finding indicates that statherin is a protein present in the first and final minutes of AEP formation with a similar abundance, suggesting that this protein is not highly susceptible to proteolytic degradation as histatins when bound to enamel; or this protein is not replaced by other proteins or peptides that are incorporated to AEP. However, our previous *in vivo* AEP peptidome study identified and characterized five naturally occurring statherin peptides ranging from N-terminal to C-terminal, demonstrating the presence of statherin peptides [22]. Similar phenomenon was observed with Cystatin D, where there was no significant abundance change according to the progress of AEP development. Other cystatins, such as cystatin S and SA demonstrated a different comportment, where these two proteins relatively increased in 10 and 60 minutes AEP formation, and relatively reduced in the last time period assessed.

Unexpected and interesting behavior was observed with acidic PRP1, a phosphoprotein that present a high affinity for hydroxyapatite and is potent inhibitor of secondary calcium phosphate precipitation, which is in large part due to their two phosphate groups linked covalently to Ser residues in position 8 and 22 [28]. This phosphoprotein showed a relative increase of 137% after 120-min AEP formation (Figure 3D). This observation is quite important since the predominant role of this protein family in the oral cavity is believed to be related to mineral homeostasis and the maintenance of tooth integrity.

Well-characterized salivary proteins such as amylase, MUC5B, lysozyme, and lactoperoxidase demonstrated a significant increase change according to the development of *in vivo* AEP (Figure 3E, F, G, H). All these proteins present protein-protein interaction features with other salivary proteins [20], [21]. This characteristic can justify the relative increase in the level of these proteins in the last stage of AEP formation, where for example these proteins can link to other proteins such as histatin 1. In this relation, recently we demonstrated that histatin 1 are able to interact with a total of 43 salivary proteins, including, amylase, MUC 5B, lysozyme, and lactoperoxidase [29].

In summary, this is the first study that investigates the dynamic process to the AEP formation by using proteomic approaches. In addition, this study demonstrated that there is a tendency for salivary proteins with affinity to calcium and phosphate be more abundant in the early stages of the AEP formation while proteins with recognized protein-protein interaction property is more significant in the final development of the AEP. Understanding pellicle formation is of great interest in the field of preventive dentistry due to pellicle serving as a solid support for the development of the plaque biofilm. Thus, it is reasonable to postulate that interference in the protein composition and structure of AEP during its formation could be a significant preventive approach. In the long term, these findings could be used to develop salivary substitutes and therapeutics for the control of biofilm growth and remineralization of early caries.
